# Fostering Patient-Clinician Communication to Promote Rapid HIV, Hepatitis B Virus, and Hepatitis C Virus Diagnostic Testing: Conceptual Development of a Multilingual App

**DOI:** 10.2196/49251

**Published:** 2023-11-16

**Authors:** Carter Brown, Guillaume Roucoux, Olivia Rousset-Torrente, Saleh Ali, Lisa Yombo-Kokule, John Chaplin, Olivier Chassany, Martin Duracinsky

**Affiliations:** 1 Épidémiologie Clinique et Évaluation Économique appliquées aux Populations Vulnérables (UMR-S 1123) Université Paris Cité Paris France; 2 Unité de Recherche Clinique en Economie de la Santé General Administration of Public Assistance of Paris Hôpital Hôtel-Dieu Paris France; 3 Institute of Health and Care Sciences University of Gothenburg Centre for Person-Centred Care Sahlgrenska Academy at Gothenburg University Gothenburg Sweden; 4 Service de Médecine Interne et d'Immunologie Clinique Hôpital Bicêtre General Administration of Public Assistance of Paris Le Kremlin,-Bicêtre France

**Keywords:** app development, agile development, mobile health, mHealth, user-centered design, communication barriers, migrants, HIV, AIDS, hepatitis, rapid diagnostic testing, public health

## Abstract

**Background:**

Migrants are disproportionately affected by HIV, hepatitis B virus (HBV), and hepatitis C virus (HCV). Clinicians, at times, fail to offer rapid diagnostic testing (RDT) for these viruses when a language barrier exists in the patient-clinician relationship, therefore creating missed testing opportunities. Although their effectiveness has been demonstrated elsewhere, conventional, in-person interpreters are costly and underused in practice. Furthermore, clinicians often call upon ad hoc interpreters, which introduces complexities in the clinical relationship. Digital solutions exist to diminish the burden of language barriers; however, the challenges of developing a multilingual and multicultural app have yet to be documented with respect to RDT in the nonfrancophone migrant population in France.

**Objective:**

Our goal was to design a multilingual app to overcome language barriers, health literacy barriers, and fears related to being tested to promote RDT of HIV, HBV, and HCV in the nonfrancophone migrant population in France.

**Methods:**

A combination of qualitative methods, agile development, and user-centered design was used. We conducted 2 focus groups (FGs) with 12 participants, including physicians, nurses, and social workers conducting RDT, as well as 1 modified Delphi survey with 68 participants including physicians and nurses. FGs explored the content (risk factors and medical history), functions (cultural adaptation and instant translation), and interface ergonomics (graphics and font) needed in the app. The Delphi presented 95 content items that the researchers sought to include in the app.

**Results:**

Using FGs to inform the Delphi survey, we scientifically determined the app’s content consisting of 95 items using expert consensus, developed a mock-up, and conducted initial user testing. We created an app that contains both migrant and clinician interfaces and includes a sociodemographic, risk assessment, health literacy, and testing barrier questionnaires available in 11 languages. Educational content is related to HIV, HBV, and HCV, along with the ability to understand whether the migrant agrees to be tested.

**Conclusions:**

This study allowed us to conceptualize a multilingual app that aims to increase the acceptance of RDT for HIV, HBV, and HCV. The specific features of the Assistant intelligent au dépistage des allophones app were designed to overcome the testing barriers in the nonfrancophone migrant population. The next phase will be an implementation study, as we intend to validate our app.

## Introduction

Migrants are disproportionately affected by HIV, hepatitis B virus (HBV), and hepatitis C virus (HCV). A meta-analysis comparing migrants with native-born populations found that migrants have a high HIV prevalence ratio [1]. A recent French study found that among migrants living with HIV, HBV, or HCV, 56%, 71%, and 89% were respectively diagnosed in France [2], whereas another study reported that 57% of migrants who are HIV positive acquired the virus while in the country [3]. Regarding HBV and HCV, it has been documented that patients who are HIV positive are at a high risk of coinfections [4]. Thus, health education and screening are important public health actions that must be better developed to protect, empower, and enable the migrant population in France.

In 2017, the French Office of Immigration and Integration (OFII) implemented optional rapid diagnostic testing (RDT; results within 20 min) for HIV, HBV, and HCV during mandatory medical consultations, which each migrant attends as part of the administrative procedure to obtain their residence permit. These RDTs are conducted with a few drops of blood collected from a finger prick. Since the implementation of these RDTs, our research team has introduced a risk assessment questionnaire to be answered by the migrant and has been collecting data about the acceptability to be screened and, when denied, reasons for refusal via the STRADA study [5]. Clinicians offer RDT to migrants who are aged at least 18 years. The STRADA study found that clinicians offered RDT to 83.3% of all migrants and that for all offers, 48.7% of migrants accept the offer to be screened. Thus, the overall nonacceptability of migrants to be screened was 51.3% [6]. Communication discordance was reported in 59.2% of the cases, 93.6% of which was related to language barriers. This study demonstrated that language barriers, therefore, generate substantial missed testing opportunities (MTOs) in the migrant population in France.

In 2021, OFII conducted medical consultations with migrants from 146 different countries. Although we do not have data about the languages spoken by migrants, we can imagine as much diversity in the culture of and language and dialects spoken by migrants as the nationalities represented. This diversity is a crucial element to be considered in the acceptability of RDT. A French study published in 2019 found that 57% of the men from sub-Saharan Africa acquired HIV upon their arrival in France [3]. Another study estimates that deaths from chronic viral hepatitis will surpass the mortality from HIV worldwide by 2040 [7]. Expanding RDT as an effort to detect and treat HIV and hepatitis in vulnerable populations, including migrants, is necessary to limit the spread of these infections.

Despite access to free testing and treatment regardless of income or migration status in France, migrants still face the issue of late diagnosis. Some reasons for late diagnosis have been suggested to be cultural taboos and lack of knowledge about HIV [8,9]. Language-relevant information and culturally adapted assessment methods will help more people to avoid late diagnosis. This has obvious benefits, both for the individual (eg, early access to care is linked to increased life expectancy) and the community (eg, reduction in transmission) [5].

Although their effectiveness has been demonstrated elsewhere, conventional interpretation methods such as in-person or over-the-phone professional interpreters are costly and underused in clinical situations by health professionals (HPs) [10-12]. The only alternative available to the clinician has been to call upon ad hoc interpreters, who are informal and untrained. These ad hoc interpreters might be family members, friends, or fellow compatriots of the patient (The National Council on Interpreting in Health Care, unpublished data, October 2008). Our previous study has observed both the migrant and HP calling upon ad hoc interpreters to assist the migrant in establishing communication concordance with the HP. Ad hoc interpreters, however, are subpar solutions compared with professional interpreters despite their convenience, as they are prone to committing errors while also introducing complexities in the clinical relationship such as reinterpretation of what has been communicated, creating a barrier to disclosure, and confidentiality issues [13]. Another alternative to the conventional interpretation services could be a cost-effective, instantaneously available communication tool that would overcome language barriers and diminish MTOs.

The benefits of mobile health (mHealth) solutions have been widely documented; however, the process of developing a multilingual and multicultural app have yet to be documented in the francophone life cycle of RDT. Our goal was to develop a multilingual app to decrease MTOs by overcoming language barriers to promote RDT of HIV, HBV, and HCV to nonfrancophone migrants in France. We aimed to develop a dual-interface app that will have a multilingual interface for migrants and an interface in French for HPs. We have planned to include content that will help the migrant understand the risks of the 3 viruses in question, along with the benefits of being screened for these viruses.

Knowledge about HPs’ expectations, along with end-user involvement, is fundamental to the development of new technology and plays an important role in determining how easily and quickly it will be adopted in practice [14,15], and therefore, we have engaged end users throughout the development process.

We chose to study RDT because the frequency of use in the migrant community does not coincide with the expectations of the French guidelines published in 2009, which proposed RDT as an alternative to traditional serological screening [16]. Furthermore, RDT is widely available in public, private, and community health care settings in France [17].

## Methods

### Study Design

**Figure 1:**
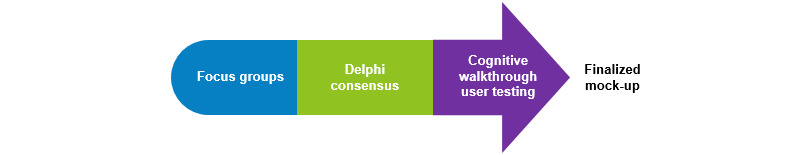
Methods used to develop and test the mock-up.

This study was part of the ApiDé study, the protocol of which was published elsewhere [18]. The sequential design of this study started with focus groups (FGs) with HPs, a Delphi consensus process, and finally cognitive walk-through (CW) of user testing, allowing each step to inform the next (Figure 1). The entire project was conducted between April 2021 and November 2022 in metropolitan France.

### Ethical Considerations

The study was approved by the French Ethics Independent Committee (Comité d’Evaluation Ethique de l’Inserm; institutional review board number 00003888; appraisal 21-768). Participants were not offered any compensation for their participation.

### Participants and Procedures

Throughout each phase of this 3-stage study, we sought participant diversity in terms of age, sex, profession, and geographic background. HPs were solicited for all 3 stages of this study, whereas migrants were solicited in the CW user testing.

#### HP Participants

HPs from the public or private health sectors, including nongovernmental organizations (NGOs) with experience of working with nonfrancophone migrant populations who were willing to participate in our study were eligible. We sought diverse sampling using a convenience sampling method by recruiting both male and female physicians, nurses, and social workers who we have worked with on other projects with a range of experience of conducting RDT for HIV, HBV, and HCV with migrants; sex workers; and lesbian, gay, bisexual, transgender, queer, and other populations who do not necessarily speak French. The exclusion criterion was not having the credentials, via medical training or a specific certification, to conduct RDT and thus a lack of experience in conducting RDT.

#### FGs With HPs

Overall, 2 semistructured FGs with HPs were conducted via Zoom (Zoom Video Communications) amidst the COVID-19 pandemic. Each FG was audio and video recorded via Zoom. Informed consent was obtained verbally at the beginning of each FG. An interview guide based on a literature review investigating health communication apps, previously published by our research team [19], was used to guide the discussion of content, screening context, user and interface design, and technical functions for a future app. Our objective was to understand the minimal content and functions needed to offer and conduct RDT with nonfrancophone migrants. Data were collected according to the COREQ (Consolidated Criteria for Reporting Qualitative Research) guidelines [20]. Owing to feasibility constraints, we were unable to include migrants in this stage of the development. However, the interview guide used was created based on migrant interviews (awaiting publication) on the same topic, which were conducted in a preceding research project.

The interview guide was divided into three sections: (1) the problems that professionals encounter in the context of rapid screening in the presence of a language barrier, (2) what professionals need to successfully facilitate rapid screening, and (3) the hypothetical app including design features.

**Figure 2:**
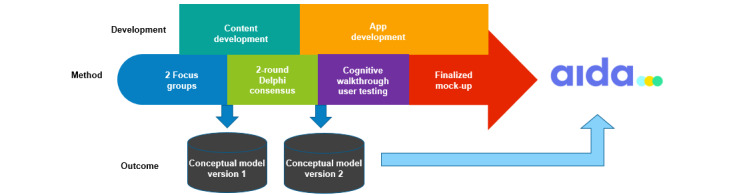
Overall process of the study, resulting in the mock-up. HP: health professional.

The FG data were analyzed using a thematic analysis technique. The data were transcribed verbatim, organized into categories, and then analyzed, allowing themes to be extrapolated from the data. This led to the construction of the first version of the conceptual model, which includes a migrant and HP interface (Figure 2).

#### Modified Delphi Survey

The modified Delphi survey allowed us to finalize the content to be included in the app, which gave rise to the second version of the conceptual model (Figure 2). Overall, 2 rounds were conducted, with 1 month between each round. Snowball recruitment process was used. We do not know the exact number of participants who were initially solicited; however, we estimate this number to be >250 individuals. This is because, for certain institutions (NGOs, OFII, and Les Centres gratuits d’information, de dépistage et de diagnostic), we were unable to obtain the complete mailing lists of HPs and thus had to rely on the directors to forward our solicitation email. Furthermore, individuals were asked to share the invitation with their colleagues.

We sought diverse sampling by soliciting both male and female physicians, nurses, and social workers based on their experience in conducting RDT for HIV, HBV, and HCV. A threshold for consensus was set for participant responses before launching the survey: the content would be accepted (average score ≥6.5); be rejected (average score ≤3.5); or owing to lack of consensus, be included in a subsequent round (average score between 3.6-6.4). The average response for each item was calculated. All but 6 items were accepted, none of the items were rejected, and 6 items did not obtain consensus. After the first round, 4 researchers (CB, LY-K, ORT, and SA) individually reviewed all the comments and then proposed new content or formulations for the 6 items that were the objects of the second round. Although each comment was reviewed, only comments that appeared more than once were considered by the researchers because of the large volume of comments obtained. Each round lasted 5 weeks. This modified Delphi expert consensus survey was derived from the papers by Hou et al [21] and Beiderbeck et al [22] on Delphi techniques.

#### Migrants

Migrants were sought from 5 OFII centers located in different regions of France (Paris, Lyon, and Nice) to increase the diversity of their backgrounds. Inclusion criteria were being aged at least 18 years, ability to speak English, and willingness to test the mock-up in the presence of a researcher.

#### The CW Method

CW is a method in which the end user explores and uses an app and commentates their experience in the presence of a researcher. This method was chosen to observe users as they discovered and explored the 2 interfaces of the mock-up as if it were in real conditions [23,24]. The objective of CW testing was to obtain user feedback about the user interface and user design of the app, which could be communicated with the development team for improvement. This approach provided instantaneous feedback while remaining in close proximity with the participant. The researchers sought to record participant reactions, criticisms, comments, and questions related to the content and usability of the app. HPs at OFII and migrants attending their medical visit were solicited to test the respective interfaces.

Overall, 3 researchers (CB, LY-K, and SF) approached migrants in the waiting room at OFII who self-identified as English speakers to ask if they would be willing to participate. The HPs included nurses and physicians working at OFII. The researchers explained the research project and the objective of the app being developed. Informed participant consent was obtained verbally before each test. CW was conducted in a dedicated, private office in the clinic, where only the migrant or HP were present with the researcher. Participants were asked to freely use the app and to freely express themselves aloud with any questions, comments, or concerns while the researcher sat next to the participant, noting the remarks made by the participant.

Then, 4 researchers (CB, LY-K, SF, and ORT) independently reviewed each comment noted from the user testing while taking notes themselves. All the notes were compiled into 1 document, and then, each researcher independently reviewed all the results. The researchers met to discusses the notes, comments, and eventual changes that should be made. Once consensus was obtained, changes and corrections were communicated with the developers who modified the mock-up accordingly, which gave rise to a finalized version. We referenced the updated mHealth app trustworthiness checklist by van Haasteren et al [25] as a proven, valuable resource for the development of a trustworthy app.

## Results

### Focus Groups

#### Sociodemographic Data of Participants

In total, 12 HPs fitting the criteria participated in the FGs: a nurse (woman: n=1, 8%), physicians (women: n=5, 42%), and social workers (women: n=4, 33%; men: n=2, 17%), with a mean age of 44 (SD 10.7) years. Of the 12 participants, 10 (83%) spoke French as their native language. One participant self-identified as transgender, whereas another participant self-identified as not being heterosexual. Of the 12 participants, 5 (42%) self-reported that they are capable of using English in the context of RDT. Of the 12 participants, 10 (83%) participants underwent specific RDT training, whereas 2 (17%) did not but were able to conduct RDT because of their previous medical training. Participants reported conducting RDT in public hospitals, OFII, other state-sponsored testing sites (ie, Les Centres gratuits d’information, de dépistage et de diagnostic, French for free information, screening, and diagnosis center for viral hepatitis and sexually transmitted infections), nonprofit organizations, private establishments (eg, dance clubs, sex clubs), social housing complexes (eg, group homes, reception facilities), and public spaces using mobile testing units and kiosks.

#### Thematic Analysis Results

##### Overview

**Figure 3:**
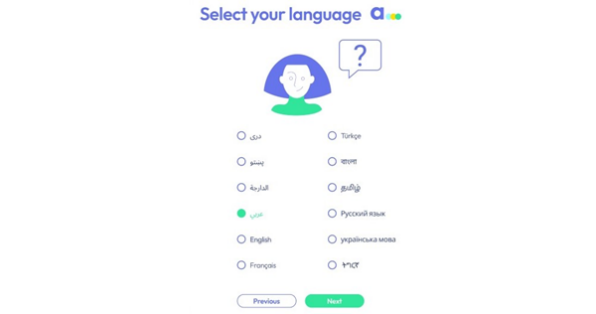
An example where the participant could not select their option. The mock-up was only in English; therefore, the unaided cognitive walk-through had to be interrupted at this point.

FGs were transcribed verbatim and then analyzed thematically by 3 researchers (CB, GR, and MD) using NVivo (version 11; QSR International). Each researcher’s codes were triangulated by the research team for consistency and validity of results. Overall, 9 themes emerged from the thematic analysis, resulting in the definition of the most important themes that must be included in the app to successfully conduct RDT (Figure 3). Comments with respect to visual aspects such as color, font, and images were also compiled to help inform the user interface design of the app. Overall, HPs believed that an electronic tool would improve their RDT practices and were, consequently, supportive of its development. Many current practices and subsequent problems were expressed along with participants’ ideas to address these problems. Participants discussed the methods they use to approach the subject of RDT, different advice they give to migrants, tools used to help communicate when there is a language barrier, and other factors that help in their practice. Participants also spoke about the topics that should be discussed or avoided with the migrant in lieu of RDT, need for cultural adaptation, and potential content and hypothetical features of our future app.

##### Problems Related to Language Barriers and Language Interpretation

Although language barriers were central to discussions about difficulties during consultations, participants spoke about short consultation times in general:

Most screenings are conducted in 10 or 15 minutes. Uh, afterwards, there are people with whom it takes longer when things emerge that really need more time.

Social worker; female

RDT is optional and, therefore, not the main objective for HPs or migrants during consultations. Before even being able to offer an RDT, some migrants need to be informed about what these tests are examining and the potential implications (ie, a positive result), according to participants. There is a need for health education and, therefore, HPs spoke about the essential information that needs to be shared with the migrant before offering an RDT:

It’s true that it’s not easy to explain what we offer [to the migrant] and it’s really necessary to come back to health education, to explain diseases, to explain viruses, to explain how they are transmitted, to explain diseases that are transmitted by sexual means and I find that it takes me a lot of time because there are often people who don’t know what a sexually transmitted disease is and therefore it’s not always easy to explain all that. There is really a whole speech needed based on explanations before [migrants] can give their consent to do this type of test.

Physician; female

RDT requires a brief consultation and is comprehensive at the same time to cover the copious amounts of information that is needed to be shared with the migrants. This already poses a major challenge for HPs, which is further complicated by the presence of a language barrier.

Overall, 67% (8/12) of the professionals spoke about a telephone interpretation service Inter Service Migrants Interprétariat at their disposition. This service is costly, and not all participants have access. Among the participants who have access, not all of them knew how to use it, and others intentionally choose not to use it because of long wait times. A professional stated the following:

...For us ISM is very expensive, and it’s a real problem.

Social worker; male

Another participant spoke about the essential information that needs to be appropriately communicated with the migrant; however, migrants are often left with incomplete information:

Exceptionally we will take the time to use ISM and explain [RDT] to the person but unfortunately [migrants] are screened with very partial information of what the screening includes, and then for the results...to explain the difference between serological and antigenic and/or antibody testing...that a TROD is only valid for a limited time...explain that this TROD has tested one disease but not another...explain the importance of protecting oneself, etc. It’s really this information that must appear in these kinds of applications.

Physician; female

Owing to long wait times in accessing a professional interpreter, a professional stated the following:

[We resort to] translation with certain Internet sites which we use to get by because we have very little money.

Midwife; female

The limitation of the existing translation services previously described in the literature have the potential to create errors in practice.

##### HPs’ Need to Successfully Facilitate Rapid Screening

Participants spoke about their need to create a safe, confidential environment during the consultation, so that the migrant feels at ease. This includes educating the migrant about RDT and its implications. Participants spoke about the need for simple, nontechnical, communication:

The number one goal for this kind of application would be to provide information that is clear and easily understood.

Physician; female

Along the same lines, participants spoke about the volume of information that should be communicated during the consultation and how getting into the fine details related to RDT was a difficult and time-consuming task with migrants who are not francophone. A participant added the following:

All the little details are what can make the interview heavy. And to have a little bit of simple language, simple, simple, to make people understand things. That’s really what we need to avoid getting into complicated things...it’s confusing, especially...often people don’t have any information.

Social worker; male

To offer and successfully perform an RDT with informed consent, certain information should be obtained and shared with the migrant. This includes sociodemographic information and assessing the potential risks the migrant might have been exposed to. Participants gave insight into what information needs to be communicated with the migrant, including how to speak about both positive and negative RDT results:

I have always tried to convey the fact that it was a chance for them to have been screened because it allows them to access care.

Physician; female

##### The Concept of an App: Design Features

The researchers projected various fonts and a large color palette to invite participants to imagine the design of a future app. Without hesitation, participants seized the opportunity to express their opinions in terms of interface design (user experience; UX). While looking at several fonts, a participant said the following:

I would say more rounded letters rather than very pointed ones.

Physician; female

Another participant added the following:

It has to be really big to be read...not too small...and then you have to be wary of fonts with other alphabets [ie, Arabic and Japanese].

Nurse; female

Simple and visible design features are necessary support positive usability of the app.

In terms of graphics used to support written content, participants favored visuals to support the written text in the app, insisting on simplicity:

It has to be simple...and easily understandable...it’s true that maybe little diagrams or drawings like a comic strip can sometimes explain the text in a simpler way.

Physician; female

Colors and the overall theme of UX were also discussed. Globally, participants agreed that the app should be “calm” and “pleasant.” A participant suggested to “avoid red...blood...red, [because it represents] anger” (physician; female), whereas another suggested “blue because it’s pleasant...and it’s not worrisome” (nurse; female).

FGs allowed us to obtain the minimal content themes and design features needed in an app to successfully offer and conduct RDT. This information became the object of the first version of our conceptual model (Figure 2).

### Content Development and Delphi Consensus

#### Content Development

**Figure 4:**
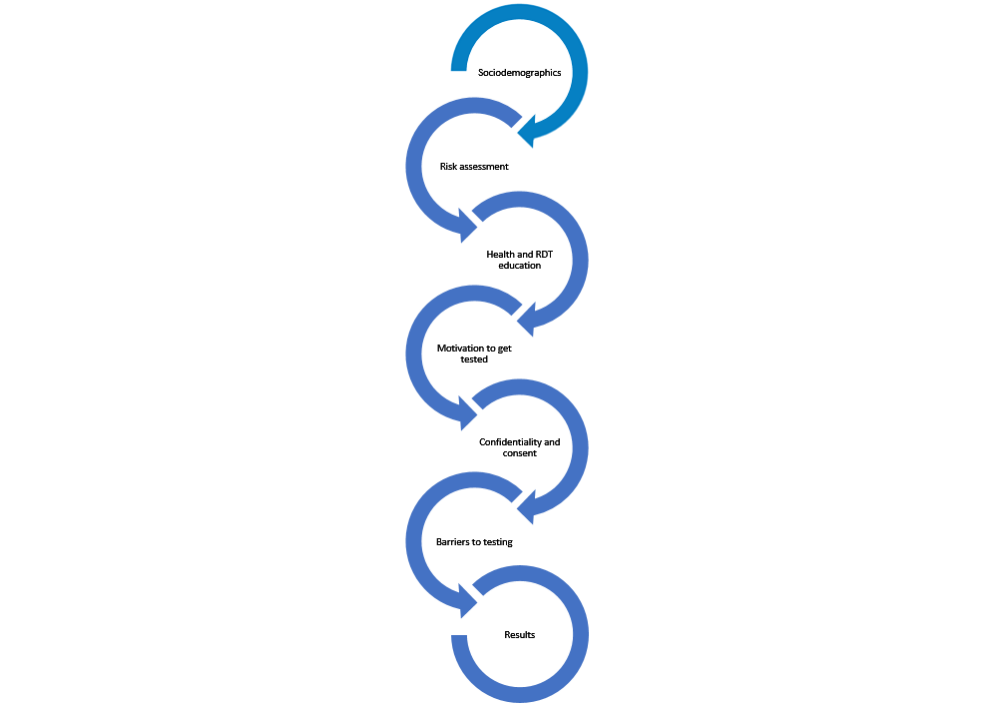
Most important content to be included in the migrants’ interface in the conceptual model version 1. RDT: rapid diagnostic testing.

To enrich the content that we sought to include in the app, we obtained screening questionnaires and educational information used by different NGOs and public institutions that conduct RDT to create a pool of content that corresponded to the first version of our conceptual model. We also investigated the existing tools for inspiration in terms of UX and content. Overall, 4 members of the research team (CB, MD, OTR, and SF) gleaned and classified the content obtained into the 9 themes that emerged from the FG as necessary information to obtain or share with the migrant: *sociodemographics, risk assessment, health education, RDT education, motivation to get tested, confidentiality, consent, barriers to testing,* and *communication of test results* (Figure 4). We also included a validated health literacy assessment [26]. The entire content was sized down by the research team based on relevance and overall comprehension.

#### Modified Delphi Consensus

The entire content was transformed into a 95-item questionnaire, which was the object of the modified Delphi survey. Overall, 68 participants responded to the first round. Of the 68 participants, 3 (4%) participants’ responses were removed from the data analysis because they reported a lack of experience in conducting RDT with nonfrancophone migrants and therefore did not complete the questionnaire. Responses from 96% (65/68) of participants were analyzed (Table 1). Consensus was obtained for 89 items (approved), with 6 items in discordance. No item was rejected. A total of 474 comments were made by participants, among the 95 items. These comments included different phrasal formulations, remarks about the pertinence of the item, personal opinions, and suggestions for different items that could replace the proposed item.

A total of 25 participants responded in the second round. Compared with the first round, the participants in the second round included only women (25/25, 100%), and most (23/25, 92%) were working in the public sector (Table 1). Consensus was obtained for all 6 items (approved), and 23 comments were made. The same 4 researchers once again reviewed each comment and came to an agreement on the entire 95-item list of content to be included in the app. The main themes along with example content can be found in Table 2.

**Table 1:** Delphi participant demographics.

Characteristics		Round-1 participants (n=65), n (%)	Round-2 participants (n=25), n (%)
**Sex**			
	Male	12 (18)	0 (0)
	Female	53 (82)	25 (100)
**Profession**			
	Nurse	31 (48)	14 (56)
	Physician	27 (42)	10 (40)
	Social worker	1 (2)	0 (0)
	Other	6 (9)	1 (4)
**Professional sector**			
	Public	46 (71)	23 (92)
	Private	9 (14)	1 (4)
	Nonprofit	10 (15)	1 (4)
**Attended formal RDT^a^ training**			
	Yes	46 (71)	12 (48)
	No	19 (29)	13 (52)
**Frequency of conducting RDT (per month)**			
	1-2	26 (40)	9 (36)
	>2	39 (60)	16 (64)
**Experience in conducting RDT (years)**			
	<1	11 (17)	10 (40)
	1-5	42 (65)	15 (60)
	>5	12 (18)	0 (0)

^a^RDT: rapid diagnostic testing.

**Table 2:** Themes and example content.

Theme	Description of the theme	Example content
Sociodemographics	Questions to obtain personal information including age, native language, and marital status	“What year were you born?”
Risk assessment	Questions to assess any risks the individual might have been exposed to, including the number of sexual partners, drug consumption, and medical history such as having had an operation or received a blood transfusion	“In the last 12 months, how many sexual partners have you had?”
Health education content	Educational information including the definition of viruses being tested, how these viruses can be contracted, and what treatment options exist	“How is hepatitis B treated?”
RDT^a^ education content	Educational information about what RDT is, how they are conducted, and reliability of the results	“How does a RDT work?”
Motivation to get tested	The reason why the migrant chose to be tested	“These viruses are frequent in my home country.”
Confidentiality	Information about the nature of the testing and results	“This consultation is confidential and the information exchanged will not be shared with anyone.”
Consent	A question to obtain informed consent to be tested	“Do you want to be tested?”
Barriers to testing	If the migrant chose not to be tested, the reason for the rejection	“I’m afraid of losing my family.”
Test results	The results of the RDT and what they might imply	“Your test for the hepatitis C virus is positive. These results are preliminary and therefore an IV blood draw is needed to confirm the result. We will schedule an appointment together for confirmatory testing.”

^a^RDT: rapid diagnostic testing.

### CW Results

Following a series of sprints—defined time slots when the research and development team worked together on specific objectives—over the course of 6 months, the research team received a semiclickable mock-up in June 2022. The mock-up includes both patient and HP interfaces along with an administrative interface for the purpose of a future quantitative accessibility study. It was developed using Figma (Figma, Inc), a collaborative web-based prototyping tool for interface design. The objective for the mock-up was to develop a progressive web application to be used on a tablet in portrait mode.

The patient and HP interfaces were both tested using the CW method. The migrant interface was developed and tested in English with anglophone migrants, whereas the health care provider interface in French was tested with francophone participants. The researchers explained the app to the participants and asked them to use it while verbally expressing their comments, questions, and concerns. During this process, the researchers intervened only when necessary; otherwise, they remained as silent observers, taking notes of all the remarks made by the participant. However, at times, the semiclickable nature of the mock-up required explanation and directive. For example, the participant could not select the language on the screen that asks the user to choose their language. The mock-up included only the English version of the content; thus, the researcher had to explain this aspect (Figure 3).

We tested the patient interface in person with 23 migrants in 4 different OFII centers. The clinician interface was tested with 8 clinicians from 5 different OFII centers. Participants were asked to freely use the app and to express themselves aloud with any questions, comments, or concerns while the researcher sat next to the participant, noting the remarks made by the participant. Participants expressed their opinions about the content, including the visual aspects of the app; asked questions if the meaning of the content was not clear; and gave their overall impression about the app—including its usability. The researchers took notes and then compiled the participant feedback and questions in 1 document to compare and contrast the entire CW results. A total of 277 content and design remarks were made by the participants.

**Figure 5:**
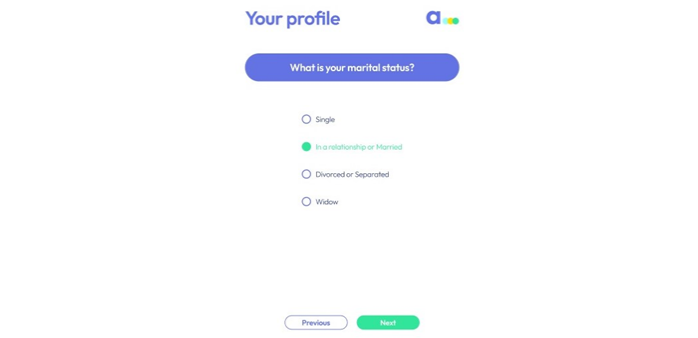
Screenshot of a screen where participants made suggestions.

To give an example related to content, 9% (2/23) of the participants suggested that we add the option “separated” to the “What is your marital status?” question (Figure 5).

**Figure 6:**
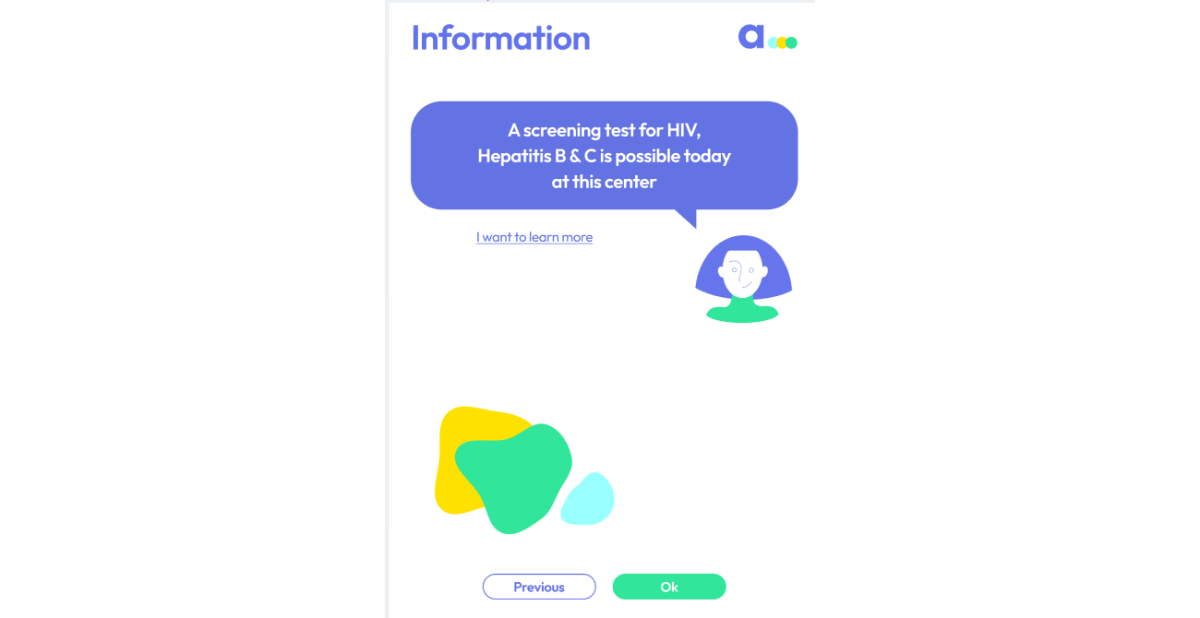
A screen that resulted in several comments. The decision to change option Next to Ok was made to make this screen more coherent.

Figure 6 shows another example of comments from the screen that reads, “A screening test for HIV, HBV, and HCV is possible today at this center.” In total, 4 separate reactions were recorded by the researchers including the suggestion to change “Next” to “Ok” or “I understand” (3 times), so that participants better understood the main content on this page. A participant asked whether the tests were conducted using a urine sample, prompting the investigators to consider placing practical information about RDT before this screen in the user journey of the app.

**Figure 7:**
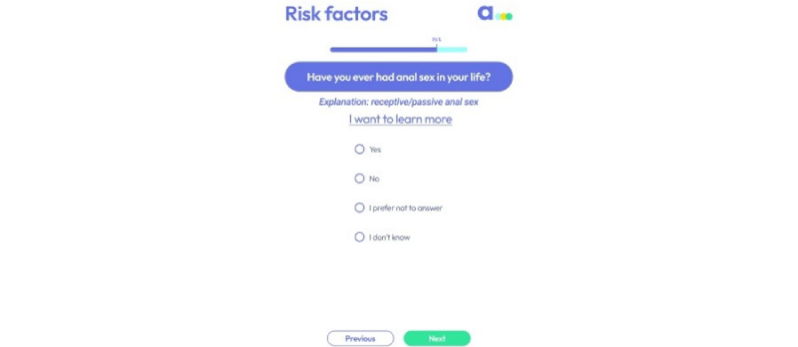
A screen that resulted in several comments during user testing.

A risk assessment question rendered several remarks. The question asks users, “Have you ever been penetrated anally?” (Figure 7). A participant suggested that “penetrated anally” be changed to “anal sex.” Another participant said that the question was “vulgar,” and 9% (2/23) of the participants expressed cultural concerns. A participant said, “I don’t think Muslims will like this section,” whereas another participant stated, “religious or far right conservatives might not like this.”

Overall, both migrants and HPs were in favor of using the app and found it useful. A participant said, “I like it. The language is friendly, not so academic. It’s cool. It will help a lot. It’s quick, too!” whereas another participant said, “The application is intimate...good idea.”

User testing questioned the critical design decisions and content, ultimately allowing the researchers to improve the app. In contrast, HPs found the app to be useful but worried about how it would work in their daily practice without taking a lot of time. The research team studied all the comments and remarks and decided upon changes. The ensemble of modifications was communicated with the app developers, who then finalized the mock-up.

## Discussion

### Main Outcome

We developed, designed, and tested a dual-interface app to promote rapid screening in France. The app will be able to obtain migrant sociodemographic information, along with having the capacity to conduct a risk assessment and a short health literacy questionnaire in the migrant’s native language. Migrant responses will be translated to French and communicated to the HP. Furthermore, there will be rapid screening and health educational content, which explains how RDT is conducted, its purpose, and limits. We have also included information about the health care system in France pertaining to the 3 viruses in question. The design of our patient-centered app empowers the migrant to independently report their personal information and has the capacity to obtain informed consent or disapproval to be screened. Empowering the patient to communicate and respond to questions about personal and, at times, intimate information in their native language could lead to great involvement and engagement as Hickmann et al [27] have demonstrated in personal health care. Meanwhile, being able to efficiently communicate the patients’ responses to the HP can help the professional to adapt their discourse to the particular patient in front of them.

The app is intended to be used, first, by the migrant in the waiting room, for example, before interacting with the HP. This could help alleviate the time-strapped consultations. It is probable that HPs will rely less on both professional and ad hoc interpreters to propose an RDT.

For the migrant interface, the user will select their native language from 11 languages and then be asked a series of sociodemographic and risk assessment questions. There is also a short health literacy assessment along with educational content about the viruses being tested, the RDT itself, prevention, and treatment and health care coverage information in France. The migrant will have access to a summary of their responses, in addition to having the capability to type questions in their native language for the HP. This interface is to be used only by the patient before meeting the HP.

Once the migrant is with the HP, the migrant will provide the HP with a unique ID that will allow the HP to obtain a resume of the migrant’s responses in French. From this moment forward, the migrant will no longer need the app as the consultation will be conducted with the HP’s interface. If the HP needs to obtain or provide additional information to the migrant during the consultation, there will be a speech-to-text function where the HP will speak in French and the app will dictate and translate it to the migrant’s native language. The migrant will be able to respond in their native language, and it will be dictated and then translated to French for the HP.

Our app does not seek to replace the HP or professional interpreters but to empower the patient to take an active and, if desired, independent role in their decision to be screened. Design suggestions from a similar study exploring the view of patients who are deaf and hard of hearing regarding an mHealth app to foster communication with pharmacists found that although the app should be culturally and linguistically diverse, there is still a strong desire to have sign language interpreters available [28]. A recent study found that approximately half of all patients prefer that the HP has the last word in the decision-making process [29]. Thus, in terms of decision-making, the patients’ preferences depend on several factors, and therefore, the role of the HP could be crucial in determining whether the patient is screened.

### Study Strengths and Limitations

The combination of qualitative methods used in this study allowed us to engage both HPs and migrants throughout development. User engagement when developing new technology is crucial in determining the rate at which it would be adopted and its effectiveness [14,30].

The different stages of this study primarily involved HP. The representation of HP, in particular, those who responded to the second round of Delphi, was limited. In addition, although migrants were included in the previous qualitative research that informed this study, the CW method used to test the mock-up was limited to migrants who speak English. Owing to budgetary constraints, we were unable to test a multilingual mock-up, therefore limiting the migrants’ involvement in this aspect of the study. Further studies will investigate the entire content in all 11 languages.

It could have been interesting to conduct an FG with migrants to elicit their impressions about the proposed app. Perhaps, their input would have influenced some app design decisions.

Future studies should consider and investigate different accessibility features for apps. For example, a fully audio-based and speech-based response and command feature could target users who are illiterate or blind. It would also be interesting to include a zoom feature to allow users to change the font size of the content.

Very few self-assessment tools or development checklists exist for the development of mHealth resources. We chose to use the mHealth app trustworthiness checklist as it assesses our app completely; however, there were several aspects of the assessment that are not yet relevant to our app (ie, question regarding the app store). A better-adapted tool should be developed to guide the development of such mHealth tools.

### Conclusions

For an app to be used by migrants and clinicians in practice, we understand that it requires to meet the needs of both parties. We developed a multilingual app with future users, to decrease MTOs by overcoming language barriers and to promote RDT of HIV, HBV, and HCV in the nonfrancophone migrant population in France. We have developed a fully functional app, named AIDA (Assistant intelligent au dépistage des allophones), which will be the subject of a future validation study.

## Abbreviations

AIDA: Assistant intelligent au dépistage des allophones
COREQ: Consolidated Criteria for Reporting Qualitative Research
CW: cognitive walk-through
FG: focus group
HBV: hepatitis B virus
HCV: hepatitis C virus
HP: health professional
mHealth: mobile health
MTO: missed testing opportunity
NGO: nongovernmental organization
OFII: French Office of Immigration and Integration
RDT: rapid diagnostic testing
UX: user experience
